# Cold chain management practices of health care workers in primary health care facilities in Southern Nigeria

**DOI:** 10.11604/pamj.2017.27.34.11946

**Published:** 2017-05-11

**Authors:** Esohe Olivia Ogboghodo, Vivian Ossaidiom Omuemu, Oisedebame Odijie, Ofure Jennifer Odaman

**Affiliations:** 1 Department of Community Health, College of Medical Sciences, University of Benin, PMB 1154, Benin City, Nigeria

**Keywords:** Practice, determinants, cold chain management

## Abstract

**Introduction:**

Vaccination has caused a dramatic reduction in the threat of diseases that were once widespread and often times fatal. The efficient practice of cold chain management is therefore key to ensuring that the benefits of vaccination are sustained. The objective of this study is to assess the practice of cold chain management among health workers in primary health care facilities in Benin City, Edo State.

**Methods:**

A descriptive cross-sectional study design was employed in this study. The study population consisted of registered nurses, auxiliary nurses and community health extension workers in primary health care facilities in Benin City, Edo State. The tool for data collection was pre-tested, structured self-administered questionnaire. Bivariate analysis between socio-demographic variables and practice of cold chain management was done. Binary logistic regression was also done to determine significant predictors of practice of cold chain management. The level of significance was set at p < 0.05 for all associations.

**Results:**

A total of 425 respondents participated in this study. Over two-thirds of respondents 314 (73.9%) had good practice of cold chain management. Significant determinants of practice of cold chain management were cold chain management training (p = 0.004), presence of functional refrigerators (p = 0.016), NPI supervision (p < 0.001) and higher level of education (p < 0.001).

**Conclusion:**

The practice of cold chain management among respondents was fair. All stakeholders should ensure they work collectively towards ensuring that favorable environments which would improve the practice among health workers are put in place.

## Introduction

Vaccination has greatly decreased the burden of infectious diseases globally and has dramatically decreased the threat of diseases that were once wide spread and often times fatal [1]. Vaccination decreases health care cost to both patients and the health care system by decreasing the incidence of vaccine preventable diseases [1].

For vaccine delivery system to be successful, essential vaccine needs to be available and of good quality. The quality of vaccines can only be ensured by a functional cold chain system. Vaccines are highly thermo-sensitive substances which have a fixed shelf life that lose viability over time. This loss is irreversible and accelerated if proper storage and temperature conditions are not maintained [2]. Freezing or heat exposure can totally or irreversibly damage the efficacy of vaccines and increase the risk of side effects [3]. Administration of vaccines that are not potent will lead to failed immunization of the individual against vaccine preventable diseases.

For cold chain management to be efficient, three major elements are required. These include well trained personnel, reliable transport/storage equipment and efficient management procedures [4]. An absence of any of these would lead to a deficient cold chain system. Health workers play an important role in maintaining an undisrupted cold chain as they are the last point of contact between the vaccines and the recipient. Hence, it is very pertinent that they be trained and supervised regularly in order to ensure efficient practice of cold chain management.

In addition to training and supportive supervision of health workers, logistic materials and tools for monitoring storage temperature (thermometers, VVMs, temperature logging charts among others) should be available at health facilities [5]. These contribute to encouraging health workers to put into practice knowledge that has been acquired, hence, supporting effective and efficient immunization [6]. In 2011, 2.8 million doses of vaccines were lost in 5 countries due to cold chain failures [7]. The CDC has estimated that each year, 300 million pounds worth of vaccines alone are destroyed globally due to improper storage and distribution [8]. These scenarios of failure of cold chain management has an end effect of causing wastage of vaccines which can be expensive and be in short supply.Refrigerators for vaccines have reportedly being found to be used in storing other things like foodstuff, laboratory reagents and drugs in some private clinics in Lagos, Nigeria [9].

Nigeria fell short in achieving the Millennium Development Goal (MDG) 4, to reduce mortality among children under the age of 5 by two-thirds from 1990 to 2015 [10]. The under-5 mortality rate in Nigeria was 124/1000 as at 2013, which was high [11]. Over 85% of child mortality is attributable to deaths in the new born periods resulting from malaria, pneumonia, and diarrhea of which the latter two are vaccine preventable [11]. Due to the various challenges of cold chain management in Nigeria as has been highlighted above, vaccines cannot be properly stored and the potency of vaccines cannot be ensured. This has invariably contributed to the high under-5 mortality rate being experienced in Nigeria. This study was carried out to assess the level of commitment of health care workers to cold chain management in terms of their practice and the determinants of their practice, thus serving as a guide to identifying areas to focus intervention mechanisms.

## Methods

The study was a descriptive cross-sectional study carried out in selected primary health care facilities in Egor and Oredo Local Government Areas of Benin City, Edo State. There are 53 primary health facilities that offer vaccination services in both LGAs. A minimum sample size of 422 was calculated using the formula for single proportion [12]. A multi-staged sampling technique comprising 3 stages was used to select respondents.

Stage 1 was selection of Local Government Areas. Two LGAs (Oredo and Egor) were selected using simple random sampling technique by balloting from a sampling frame of the 3 LGAs in Benin City. Stage 2 was selection of wards, two wards each were selected from the LGAs using simple random sampling technique by balloting. In stage 3, all the health facilities that offered immunization services in the 4 wards selected were used in this study. All health workers in the selected health facilities who met the inclusion criteria were recruited for this study. The study population comprised Registered nurses, Auxillary nurses, Community Health Extension Workers involved in vaccination services in primary health facilities in Egor and Oredo LGAs, Benin City.

Data was collected using a pre-tested structured self-administered questionnaire comprising both open and closed ended questions. The questionnaire was divided into 3 sections. Section A sought information on the socio-demographic characteristics of the respondents, Section B sought information on respondents’ practice of cold chain management and Section C consisted of questions that assessed determinants of cold chain management. The study tool was pretested in selected primary health facilities in Ovia North-East Benin-City, Edo State and corrections were effected prior to commencement of the study. Ethical clearance was obtained from Ethical Clearance Committee, University of Benin. Written informed consent was obtained from respondents. In order to ensure confidentiality, serial numbers rather than names were used to identify the respondents. Respondents were informed that they had the right to decline participation or to withdraw from the study at any time they wished. Respondents were also informed that there were no penalties or loss of benefits for refusal to participate in the study or withdrawal from it.

The questionnaires were screened for completeness by the researcher after which they were coded, entered into the IBM SPSS version 21.0 software and analysed. Practice was assessed using a total of 7 questions. A score of ‘1’ was given for correct responses and ‘0’ for incorrect responses. The maximum achievable score was ‘7’ and the minimum ‘0’. The total score for each domain was calculated and converted to percentages and were grouped as poor practice (Scores 49.9% and below) and good practice (Scores 50.0% and above). Test of associations were carried out using chi-squared tests or the Fishers’. Exact test where appropriate and binary logistic regression was used to further determine significant predictors of practice of cold chain management. The level of significance was set at p < 0.05. Frequency tables were used to present the results.

## Results

A total of 425 respondents participated in this study. The mean age was 37.5 ± 8.7 years with the highest proportion of respondents 153 (36.0%) being in the 25-34 years age group. Three hundred and seventy-one (87.3%) were females. Three-fifths of respondents, 259 (60.9%) belonged to the Benin Ethnic group and majority of the respondents 420 (98.8%) practiced Christianity (Table 1).

**Table 1:** Socio-demographic characteristics of respondents

Variables	Frequency (n = 425)	Percent
**Age group (years)**		
15-24	20	4.7
25-34	153	36.0
35-44	152	35.8
45-54	90	21.2
55-64	10	2.4
Mean age ±SD	37.5±8.7	
**Sex**		
Male	54	12.7
Female	371	87.3
**Level of education**		
Secondary	134	31.5
Tertiary	291	68.5
**Cadre**		
Registered nurse	151	35.5
Auxiliary nurse	105	24.7
CHEW^+^	169	39.8
**Number of years of vaccine administration**		
< 1 year	35	8.2
1 – 5 years	123	28.9
>5 years	267	62.8

+ Community Health Extension Workers

Two hundred and ninety-one (68.5%) of the respondents had tertiary level of education while 134 (31.5%) had acquired secondary level of education. One hundred and sixty-nine (39.8%) of the respondents were CHEWs, while 151 (35.5%) and 105 (24.7%) were registered nurses and auxiliary nurses, respectively. Majority of the respondents 267 (62.8%) had been administering vaccines for greater than 5 years (Table 1).

Majority of the respondents 331 (77.9%) stored vaccines under appropriate conditions while 94 (22.1%) stored vaccines incorrectly. One hundred and forty-nine (35.1%) had good practice of cold chain monitoring while 276 (64.9%) exhibited bad practice. A higher proportion of respondents 247 (58.1%) and 273 (64.2%) had good practice of storing freeze sensitive vaccines and bad practice of storing heat sensitive vaccines, respectively. Over three-quarters of respondents 367 (86.4%) had good practice with regards to VVM reading. Overall, a higher proportion 314 (73.9%) of the respondents had good practice of cold chain management and 111 (26.1%) had poor practice.

Three hundred and one (70.8%) of the respondents had received training in cold chain management. Of these, 61 (20.3%), 51 (16.9%) and 189 (62.8%) had their last training less than 6 months ago, 6-12 months ago and greater than a year ago, respectively. A greater proportion of respondents 334 (78.6%) worked in health facilities that had NPI supervision. Of those that had NPI supervision, 152 (45.5%) received NPI supervisors more than once a month (Table 2).

**Table 2:** Personnel training, supervision and availability of cold chain equipment at health facility

Variables	Frequency	Percent
**Training in Cold chain management (n = 425)**		
Yes	301	70.8
No	124	29.2
**Time of last training (n = 301)**		
Less than 6 months	61	20.3
6-12 months	51	16.9
Greater than a year	189	62.8
**NPI supervision at HF (n = 425)**		
Yes	334	78.6
No	91	21.4
**Frequency of supervisor visits (n = 334)**		
< once a month	182	54.5
≥ once a month	152	45.5
**Presence of vaccine refrigerator (n = 425)**		
Yes	289	68.0
No	136	32.0
**Functional vaccine refrigerator (n = 289)**		
Yes	268	92.7
No	21	7.3
**Presence of deep freezer (n= 425)**		
Yes	171	40.2
No	254	59.8
**Presence of refrigerator thermometer (n = 425)**		
Yes	147	34.6
No	278	65.4
**Functional refrigerator thermometer (n = 147)**		
Yes	133	90.5
No	14	9.5
**Presence of temperature logging chart (n = 425)**		
Yes	190	44.7
No	235	55.3

A higher proportion of respondents 289 (68.0%) were employed in HFs that had vaccine refrigerators. Of these, 268 (92.7%) had functional refrigerators in their health facilities. One hundred and seventy-one (40.2%) and 353 (83.1%) of respondents worked in health facilities that had a deep freezer and cold boxes, respectively. One hundred and forty-seven (34.6%) and 190 (44.7%) of the respondents worked in health facilities that had refrigerator thermometers and temperature logging charts, respectively. Of those respondents employed in health facilities with refrigerator thermometers, 133 (90.5%) were in HFs with functional thermometers (Table 2).

Of those that worked in health facilities that had a deep freezer present, 161 (94.2%) worked in health facilities with functional deep freezers. All health facilities in which respondents worked had vaccine carriers 425 (100%), with majority of them 353 (83.1%) working in health facilities having less than 5 vaccine carriers. All respondents 425 (100.0%) worked in health facilities that had ice packs with a higher proportion 254 (59.8%) in those that had less than 20 ice packs. Only 130 (30.6%) of the respondents worked in health facilities that employed a cold chain technician.

The majority of respondents 299 (70.4%) worked in health facilities that had a vehicle for transporting vaccines. Three hundred and forty-two (80.5%) worked in health facilities that had access to electricity supply and electricity supply was irregular in all these health facilities. Three hundred and twelve (73.4%) of the respondents worked in health facilities that had a standby generator and of these, 286 (91.7%) worked in HFs that had a functional generator.

A higher proportion of respondents aged between 25 and 34 years 119 (77.8%) had good overall practice of cold chain management. The association between age of respondents and practice of cold chain management was not statistically significant (p = 0.270). Males had better practice of cold chain management 41 (75.9%) compared to the females 273 (73.6%). This difference in practice of cold chain management observed between sexes was however not statistically significant (p = 0.714) (Table 3).

**Table 3:** Cold chain determinants and overall practice of cold chain management

	Practice			
Variable	Good Freq (%)	Poor Freq (%)	Test Statistic	P – value
**Age group (years)**				
15-24	11 (55.0)	9 (45.0)	χ^2^= 5.172	0.270
25-34	119 (77.8)	34 (22.2)		
35-44	110 (72.4)	42 (27.6)		
45-54	67 (74.4)	23 (25.6)		
55-64	7 (70.0)	3 (30.0)		
**Sex**				
Male	41 (75.9)	13 (24.1)	χ^2^= 0.134	0.714
Female	273 (73.6)	98 (26.4)		
**Level of Education**				
Secondary	63 (47.0)	71 (53.0)	χ^2^= 73.211	<0.001
Tertiary	251 (86.3)	40 (13.7)		
Cadre				
**Registered nurses**	132 (87.4)	19 (12.6)	χ^2^= 29.834	<0.001
Auxiliary nurses	60 (57.1)	45 (42.9)		
CHEWs	122 (72.2)	47 (27.8)		
**Training in Cold chain management (n = 425)**				
Yes	209 (69.4)	92 (30.6)	χ^2^= 10.574	0.001
No	105 (84.7)	19 (15.3)		
**Time of last training (n = 301)**				
≤ 1 year ago	89 (79.5)	23 (20.5)	χ^2^= 8.119	0.004
> 1 year ago	121 (63.8)	68 (36.2)		
**Functional refrigerators (n = 289)**				
χ^2^= 5.820				
0.016				
Yes	214 (79.9)	84 (23.8)		
No	10 (47.6)	27 (37.5)		
**Temperature logging charts (n = 425)**				
Yes	187 (98.4)	3 (1.6)	χ^2^= 107.227	<0.001
No	127 (54.0)	108 (46.0)		
**Receive NPI supervisors at HF (n = 425)**				
Yes	238 (71.3)	96 (28.7)	χ^2^= 5.570	0.018
No	76 (83.5)	15 (16.5)		
**Frequency of supervisor visits (n = 334)**				
< once a month	99 (54.4)	83 (45.6)	χ^2^= 55.518	<0.001
≥ once a month	139 (91.4)	13 (8.6)		

Good practice of cold chain management was higher among respondents with tertiary level of education 251 (86.3%) compared to those with secondary level of education 63 (47.0%). This difference in practice of cold chain management observed with increasing level of education was statistically significant (p< 0.001). A higher proportion of registered nurses 132 (87.4%) had good practice of cold chain management compared to auxiliary nurses 60 (57.1%) and CHEWs 122 (72.2%). The association between cadre of health workers and practice of cold chain management was statistically significant (p < 0.001) (Table 3).

Practice of cold chain management was found to be best among respondents who had administered vaccine for less than one year 31 (88.6%), followed by those who had been administering for greater than 5 years and then 1-5 years, 226 (84.6%) and 57 (46.3%) respectively. The association between number of years of vaccine administration and practice of cold chain management was statistically significant (p < 0.001) (Table 3).

Two hundred and nine (69.4%) of the respondents who had received training in cold chain management had good practice of cold chain management compared to 105 (84.7%) of those who had had no training. The association between training in cold chain management and practice was statistically significant (p= 0.001). Good practice of cold chain management was higher among respondents who had their last cold chain management training less than one year ago 89 (79.5%) compared to those who had their training greater than one year ago 121 (63.8%).The association between the time of last training and practice of cold chain management was statistically significant (p= 0.004) (Table 3).

The practice of cold chain management was found to be better among respondents whose health facilities had a functional vaccine refrigerator 214 (79.9%) compared to those whose health facilities did not have a functional refrigerator 10 (47.6%). The association between presence of functional refrigerators in HF and practice of cold chain management was statistically significant (p = 0.016). A greater proportion of respondents who worked in health facilities that had temperature logging charts 187 (98.4%) had good practice of cold chain management compared to those who worked in health facilities that had no temperature logging charts 127 (54.0%).The association between presence of temperature logging charts in health facilities and practice of cold chain management was statistically significant (p < 0.001) (Table 3).

A lower proportion of respondents that had NPI supervision at the health facilities they worked in, 238 (71.3%) had good practice of cold chain management compared to those who had no NPI supervision at their health facilities 76 (83.5%).The association between NPI supervision and practice of cold chain management was statistically significant (p = 0.018). Good practice of cold chain management was higher among respondents who were employed in health facilities that received visits from NPI supervisors greater than once a month 139 (91.4%) compared to those who received visits from NPI supervisors at their health facilities less than once a month 99 (54.4%). The association between frequency of NPI supervisor visits and practice of cold chain management was statistically significant (p < 0.001) (Table 3).

A greater proportion of respondents employed in health facilities that had less than 5 vaccine carriers 269 (76.2%) had good practice towards cold chain management compared to those employed in health facilities that had greater than 5 vaccine carriers 45 (62.5%). The association between number of vaccine carriers in health facilities and practice of cold chain management was statistically significant (p< 0.001).

Practice of cold chain management was better among respondents whose health facilities had greater than 20 ice packs 142 (81.6%) compared to those whose health facilities had less than 20 ice packs 172 (68.5%). The association between number of ice packs present in health facilities and practice of cold chain management was statistically significant (p= 0.003). With a year increase in age, the likelihood of having good practice of cold chain management decreased by an odds ratio of 0.927 and this was statistically significant (p< 0.001, CI = 0.893 – 0.953).Males were 1.734 times more likely to have good practice of cold chain management compared to the females. This was not statistically significant (p = 0.179, CI = 0.777 – 3.869). With increasing level of education, respondents were more likely by an odds ratio of 5.267 to have good practice of cold chain management and this was statistically significant (p <0.001, CI = 2.971 – 9.338) (Table 4).

**Table 4:** Logistic regression model for determinants of practice of cold chain management

Predictors	B (regression co-efficient)	Odds ratio	95% CI for OR		P – value
			Lower	Upper	
**Age**	-0.076	0.927	0.893	0.953	<0.001
**Sex**					
Male	0.550	1.734	0.777	3.869	0.179
Female^+^		1			
**Level of Education**	1.661	5.267	2.971	9.338	<0.001
**Cadre**					
CHEWs	0.022	1.022	0.596	1.753	0.936
Registered^+^nurses/Auxilliary nurses		1			
**Number of years of vaccine administration**					
≤5 years	-2.198	0.111	0.055	0.225	<0.001
>5 years		1			
**Training in cold chain management**					
Yes	-1.531	0.216	0.111	0.424	<0.001
No^+^		1			
**NPI supervision at HF**					
Yes	-0.036	0.965	0.478	1.947	0.920
No^+^		1			

+ Reference category

R^2^= 26.3%- 38.5%, CI = Confidence Interval

CHEWs were more likely to have good practice of cold chain management compared to auxiliary nurses and registered nurses. Being a CHEW increased the chances of having good practice of cold chain management by an odds ratio of 1.022. This was however not statistically significant (p = 0.936, CI = 0.596 – 1.753). Respondents who had been administering vaccines for less than 5 years were less likely to have good practice of cold chain management. This was statistically significant (p <0.001, CI = 0.055 – 0.225) (Table 4).

Those who had had cold chain management training were less likely to have good practice of cold chain management and this was statistically significant (p <0.001, CI = 0.111 – 0.424).Respondents who received NPI supervision at their health facility were less likely to have good practice of cold chain management. This was however not statistically significant (p = 0.920, CI = 0.478 – 1.947) (Table 4).

## Discussion

All respondents who participated in the study fell within the age group of 19 to 59 years. This age range falls within the working population in Nigeria which has been stated to be from 15 to 64 years [13]. More females than males were found to have participated in this study. This may be due to the fact that a higher proportion of respondents were nurses (registered and auxiliary nurses). There is the general perception that nursing is a woman’s field stemming from old stereotypes in which women were exclusively assigned the role of being caregivers in the home, and as a result, the profession may not be attractive to men.

A higher proportion of respondents that participated in this study were community health extension workers. This is similar to a study done in Central Ethiopia in 2012 [14]. Community health extension workers are an invaluable force that drive vaccination in mostly rural and hard-to-reach communities. This is because they are mostly indigenous to these areas and can reach out to members of these communities using methods which they can easily comprehend. A higher proportion of CHEWs may therefore result in better health practices and increased immunization uptake amongst rural communities eventually culminating in a step forward towards reducing the burden of vaccine preventable diseases (VPDs) in the country and the world at large [15].

A higher proportion of respondents were of the Benin ethnic group. This may be attributed to the fact that this is the predominant ethnic group in Benin City where this study was conducted. The practice of Christianity was predominant among respondents and is a reflection of the location of the area of this study (South-south geographical zone), in which Christianity has been stated to be the main religion practiced [16].

Majority of respondents were found to have tertiary level of education. This may be attributed to the fact that the majority of respondents were either registered nurses or CHEWs. A higher level of education increases the cognitive and mental function of individuals and bestows upon them a higher capacity to translate knowledge that has been acquired into practice. This was affirmed in this study where with increasing level of education, practice of cold chain management was found to be better.

A higher proportion of respondents were found to have good practice of cold chain management. This is in contrast to findings from a study carried out in Dammam, Saudi Arabia in 2007 where majority of respondents who worked in private health centers had poor practice [17]. The success of efforts against vaccine preventable diseases is attributable in part to proper storage and handling of vaccines. Good practice of cold chain management can translate into ensuring that the full benefit of immunization is realized.

Despite overall good practice of cold chain management exhibited by a higher proportion of the respondents, more than six-tenth of health care workers had poor practice with regards to cold chain monitoring and storage of heat sensitive vaccines. This is in contrast to results from a study carried out in Kalasin Thailand in 2010 where a higher proportion of respondents stored heat sensitive vaccines appropriately and recorded refrigerator temperature twice daily [18]. Poor practice of cold chain monitoring could be due to absence of functional cold chain monitoring equipment such as thermometers and temperature logging charts in majority of the health facilities respondents worked in as was found in this study. Proper cold chain monitoring using appropriate equipment is important as vaccines are very sensitive to suboptimal temperatures. If at any time, the temperature in cold chain equipment used such as the freezer and refrigerator goes below or above the recommended temperature for vaccines stored in them, proper monitoring could detect this early and appropriate measure taken to prevent damage to the vaccines.

Majority of respondents worked in health facilities that had access to electricity however, its supply was irregular in all them. This is in congruence with findings from a study carried out in Cameroon in 2008 [19]. The abysmal state of power supply that obtains in a country such as ours may account for this finding. This could lead to exposure of the vaccines stored in refrigerators and freezers to fluctuating storage temperature. Respondents may be further discouraged from carrying out proper cold chain management as this could dull their morale to work due to the stress from having to transfer vaccines to cold boxes or another health facilities with a standby generator every time there is a loss of power supply.

A lesser proportion of the respondents were employed in HFs that had functional refrigerator thermometers and temperature logging charts. This is in contrast to findings from a study carried out in Cameroon in 2008 where majority of HFs had a temperature monitoring chart and functional thermometers [19]. This could translate into poor practice of cold chain monitoring as was observed in this study seeing as these are the principal equipment needed to monitor the cold chain.

A lower proportion of respondents received NPI supervisors at their HFs more than once a month. This may be due to location of some of these HFs in hard-to-reach areas which may discourage supervisors from undertaking regular supervision. In addition, poor accountability framework which encourages lack of discipline among supervisors may account for this finding. Poor supervision creates a workplace environment that may encourage respondents to deviate from acceptable practice with regards to cold chain management.

Of note is that respondents who had been administering vaccines for less than a year had better practice of cold chain management. This is in contrast to findings from a study carried out in Ethiopia in 2012 where respondents with more than 2 years experience were found to have more satisfactory practice and knowledge [20]. This could be attributed in part to the fact that these respondents may have had training in cold chain management less than a year ago prior to being employed and as a result, they are more likely to clearly remember details of what they had been taught. Furthermore, people who are newly employed to do a job have a lot of zeal and want to get things done quickly and efficiently in order to please their employer. This may account for the good practice of cold chain management found among a higher proportion of respondents.

The importance of cold chain management training was emphasized by the positive impact it was observed to have on the practice of respondents. Again, those who had been trained less than a year ago exhibited better practice. This further sheds light on the importance of continuous training, and reiterates the fact that training serves as a fertile ground for respondents to learn how best to convert all the knowledge they have obtained in cold chain management into meaningful practice.

The presence of cold chain equipment such as refrigerators, logging charts were also found to positively influence the practice of cold chain management by respondents. The availability of workplace equipment and infrastructure impacts on the performance of workers and quality of services they render, this may explain why workers employed in aforementioned health facilities had better practice. Adequate workplace infrastructure has also been found to improve employee productivity by 19% further supporting this finding [21].

Higher frequency of NPI visits was found to profoundly impact positively on the practice of cold chain management by respondents. This is in consonance with findings from a study carried out in Lagos in 2010, where it was found that continuous monitoring and education of health workers during supervisor visits improved their practice of cold chain management [9]. This finding in our study may be due to the fact that during NPI supervision, even as workers are encouraged to do their best, on the spot corrections of errors in vaccine management are made and these corrections would invariably lead to better practice of cold chain management and guarantee the administration of potent vaccines to the populace.

## Conclusion

In conclusion, a higher proportion of respondents were also found to have good practice of cold chain management. Cold chain management training, NPI supervision as well as availability of cold chain equipment were also found to be strong determinants of cold chain management. Managers of health facilities and health workers should work hand in hand to ensure that the integrity of the cold chain is sustained.

### What is known about this topic

- Supportive supervision improves the practice of cold chain management as was observed in a study carried out in Lagos, Nigeria between September 2007 and September 2009 in 1000 privately owned facilities.

### What this study adds

- Regular training of healthcare workers in cold chain management improves practice of cold chain management;
- Availability of cold chain equipments such as refrigerator thermometers, functional refrigerators and temperature logging charts improved the practice of cold chain management;
- More frequent NPI supervisor visits to health facilities ensured better practice of cold chain management.

## Competing interests

The authors declare no competing interest.
